# Economic gains and health benefits from a new cigarette tax scheme in Taiwan: a simulation using the CGE model

**DOI:** 10.1186/1471-2458-6-62

**Published:** 2006-03-10

**Authors:** Chun-Yuan Ye, Jie-Min Lee, Sheng-Hong Chen

**Affiliations:** 1 Department of International Trade, Overseas Chinese Institute of Technology, Taichung, Taiwan; 2 Department of Logistics Management, National Kaohsiung Marine University, Kaohsiung, Taiwan; 3 Department of Applied Economics, National Chung Hsing University, Taichung, Taiwan

## Abstract

**Background:**

This study evaluates the impact of an increase in cigarette tax in Taiwan in terms of the effects it has on the overall economy and the health benefits that it brings.

**Methods:**

The multisector computable general equilibrium (CGE) model was used to simulate the impact of reduced cigarette consumption resulting from a new tax scheme on the entire economy gains and on health benefits.

**Results:**

The results predict that because of the new tax scheme, there should be a marked reduction in cigarette consumption but a notable increase in health benefits that include saving between 28,125 and 56,250 lives. This could save NT$1.222~2.445 billion (where US$1 = NT$34.6) annually in life-threatening, cigarette-related health insurance expenses which exceeds the projected decrease of NT$1.275 billion in Gross Domestic Product (GDP) because of reduced consumption and therefore tax revenue.

**Conclusion:**

Overall, the increased cigarette excise tax will be beneficial in terms of both the health of the general public and the economy as a whole.

## Background

Particularly since Taiwan joined the WTO in 2002, the government has been taking serious measures to gain greater control over tobacco usage. The cost of cigarettes to Taiwan consumers has historically been considerably less than that to people in other countries [1,2]. On average, 77 minutes of working time is required to purchase one pack of cigarettes in India, 62 minutes in Indonesia, and 56 minutes in China; contrast this with Taiwan where a pack can be bought after only 7 to 10 minutes of work. In 2002, 4.5 million adults in Taiwan were considered smokers, or one in three [3]. Smoking-related health insurance expenses totaled around NT$20 billion per year, and an additional smoking-related loss in productivity was valued at US$1.032 billion [4]. As significant an economic burden as this had been, it was obviously worthy of policy attention. Thus, on January 1, 2002, a new tax scheme was enacted in Taiwan.

Taxation on cigarette products is universally recognized as an effective way to reduce tobacco use. In 1999, the World Bank concluded that a 10 percent increase in tobacco price would reduce tobacco use by 4 percent in developed countries and by about 8 percent in developing countries (World Bank, 1999; Chaloupka *et al*., 2000) [5,6]. Several studies have demonstrated that increased taxes have been particularly effective in reducing tobacco use among youth and young adults [7-10]. The greater effect of higher prices on youth may be a result of lower income, greater peer pressure, less concern about long-term health effects and future health costs, as well as less likelihood of being addicted. Chaloupka and Grossman (1996) used data on over 110,000 eighth-, tenth- and twelfth-grade students to examine the effect price and a variety of tobacco control policies had on youth smoking consumption. They estimated that the overall price elasticity of demand for teenage smokers was -1.31 and concluded that just over one-half of the effect elevated prices had was on the prevalence of smoking among youth. Given that tobacco use among youth is considerably more responsive to price and that most smokers take up smoking before the age of 20, significant increases in tobacco taxes should be effective in producing long-run reductions in smoking among youth.

In most countries, differential taxation rates are applied depending on filter type, cigarette length, size of the relevant factory size, or retail price. A tax incidence can be as low as 8–10 percent of the retail price of domestic hand-rolled *kretek *in Indonesia and *cheroots *in Myanmar, or as high as 85 percent for imported tobacco in Sri Lanka [11]. In Taiwan, the new tax scheme, enacted on January 1, 2002, levied a NT$16.8 (where US$1 = NT$34.6) tax excise on each packet. Under this tax scheme, cigarette tax revenues account for 40 percent of the average retail price, approximately NT$42.2 per pack. Added onto the previous NT$11.8 tax on a 20-cigarette pack was an additional NT$5 earmarked as a "Health and Welfare Tax" and 5 percent sales tax. True that a 40 percent increase would be considered high for most other products, but for cigarettes, it is still lower than the 66 percent or more in high-income countries, such as the United States [6].

In that raising cigarette tax has been proven to be highly effective in reducing cigarette consumption, a cigarette tax increase has become a preferred policy for tobacco control in many countries [12-17]. In the debate on cigarette tax policy, nevertheless, one economic counterargument is that because of the expected reduced consumption, such a tax would eventually lead to a reduction in not only tobacco production but also in tax revenues. In turn, reduced tobacco production would contribute to significant reductions in employment in the tobacco sector, including farming and manufacturing, and other related fields, such as general wholesaling and retailing. Consequently, opponents to cigarette tax increases contend that the adverse impact on the macro-economy should be considered above all else.

Once the new tax was in effect in Taiwan, cigarette consumption plunged almost 25 percent, from 46.3 billion pieces in 2001 to 34.7 billion in 2002. At the same time, the former government tobacco monopoly started to incrementally phase out tobacco production. When the Taiwan market is adjusted to meet WTO conditions, it can be expected that Taiwan tobacco leaf production will continue to fall. One market scenario is that, eventually, low-quality, low-priced tobacco will be imported from non-U.S. sources to compensate for the decreased Taiwan production [18].

An increase in cigarette taxes can be justified on several grounds. It is a relatively efficient tool for generating tax revenues, an effective approach to improving the overall state of health of the public and reducing health care costs, and an appropriate way to enhance the economic efficiency [6]. Despite the relatively low price elasticity of demand for tobacco products and the fact that taxes account for a significant share of tobacco price, even a modest tax increase would effectively increase tax revenues. Estimates suggest that in the short-run, a 10 percent increase in cigarette tax would lead to an average increase of nearly 7 percent in cigarette tax revenues [6].

As a rule, a change in the tax rate levied upon a commodity can lead to smaller, a commensurate, or a greater increase in the retail price of that commodity. In response to increased cigarette taxation, cigarette prices have, in some cases, been found to increase even more than the amount of the tax increase itself [19]. Recent studies have found, in fact, that prices in Taiwan and South Africa increased by much more than the amount of the increase in tax [20,21].

Price elasticity is a particularly important indicator since it measures the effects of a cigarette tax increase on cigarette consumption and government tax revenues. The effect of a price increase on demand depends on cigarette price elasticity – the larger the elasticity is, the greater is the reduction in consumption. Therefore, an estimation of price elasticity is a very important indicator of the extent to which a "Tobacco Health and Welfare Tax" affects cigarette consumption. Used as critical information to adjust the "Tobacco Health and Welfare Tax", price elasticity could help ensure actual policy matches explicit goals.

Using aggregate annual time-series data collected by the Taiwan Wine and Tobacco Bureau, Hsieh *et al*. (1999) found the price elasticity of demand was -0.6 for domestic cigarettes and -1.1, for imported ones. This strongly suggests that a tax increase would be more effective in reducing smoking in Taiwan than it would be in most high income countries [22]. Based on data from a national personal interview survey in 2000 specifically designed to evaluate the effect of the new tax, Tsai *et al*. (2002) obtained an average price elasticity of -0.3 for all cigarettes. This was smaller than expected since Taiwan is a highly developed country [2]. Recently, Lee *et al*. (2005) have estimated the demand for cigarettes in Taiwan by using annual time-series data and found a highly significant but negative price effect [23]. With the estimated price elasticity for domestic and imported cigarettes of -0.644 and -0.822, respectively, those authors have concluded that the tax scheme was able to significantly reduce cigarette consumption. At the same time, they claim, it can be expected that the scheme should generate additional tax revenues. There is little question that with accurate, up- to-date elasticity estimates, the effect of an increase in cigarette tax on cigarette consumption and government tax revenues can be estimated.

The counterargument that a tax increase would lead to reduced employment in many relevant sectors is easily refuted. If resources were not committed to the tobacco sector, they would readily be transferred to other productive economic activities which would in turn generate employment and tax revenues [6]. Such a shift could actually improve the economy in countries that reallocate the money spent on tobacco in order to increase the production of indigenous goods and services. Consequently, notwithstanding the immediate negligible negative impact on the macro-economy, the long-run impact would likely be positive [24].

With its aim to devise the most effective policy strategies, the government must take into account the importance of both public health and economic development. Hence, this study investigates the long-term effects of the new cigarette tax scheme in Taiwan on: (1) cigarette consumption and tax revenue; (2) tobacco-related industrial sectors; (3) macroeconomic structure and consumer welfare; and (4) health benefits.

### Consumption and tax revenues after the new tax scheme

Before January 1, 2002, the government-run manufacturer, the Taiwan Tobacco and Wine Bureau, was the sole provider of Taiwan's domestic cigarettes. From 1987 to 2002, domestic cigarettes were taxed at NT$10–11 per pack [25], whereas imported cigarettes were taxed at NT$16.6 per pack. They both were subject to an in-kind tax, i.e., a monopolistic profit, and this explained nearly 47 percent of the retail price [23]. After Taiwan's entry into the WTO in 2002, the new cigarette tax law imposed an additional tobacco tax of NT$11.8 per pack on both local and imported cigarettes. Thus, the price of local and imported cigarettes rose to NT$35.1 and NT$50.4 per pack, respectively, which represents a total increase of NT$10 for local cigarettes and NT$6 for imported ones.

As for the allocation of funds from the "Tobacco Health and Welfare Tax", there should be none 70 percent goes into a reserve fund for the national health insurance system, while the remaining 30 percent goes to the central government for tobacco hazard prevention and social welfare programs. Using tobacco tax revenues in this way not only discourages smoking, but also provides additional resources to help cover the shortage of funding in the national health insurance system.

To determine the most likely impact of "Tobacco Health and Welfare Tax" increase on the decrease in cigarette consumption and increase in total government tax revenues, in this study, we used 2001 data on price and sales figures. The average annual cigarette consumption of domestic and imported cigarettes in Taiwan was 59.3 and 67.22 packs per capita per year, respectively, for a total of 126.52 packs per capita per year in 2001 [23]. As a result, prices for local and imported cigarettes rose to NT$35.1 and NT$50.4 per pack, respectively [23]. This is a rise of NT$10 for local and NT$6 for imported cigarettes. Lee *et al*. (2005) estimated price-elasticity for domestic and imported cigarettes at -0.644 and -0.822, respectively [23]. When these values are extrapolated, authors found an increase of NT$10 for domestic and NT$6 for imported cigarettes resulted in a reduced consumption of 15.21 and 7.51 packs per year per capita, respectively, for an overall average annual reduction of 22.72 packs per person; this represents a total of 1.836 billion packs per year. Lee *et al*. (2005) also reported that the government tax revenues would increase to NT$30.849 billion from NT$22.3 billion the year before. Thus, the NT$5 increase in cigarette tax would bring in an extra NT$8.5 billion in tax revenue.

## Methods

This empirical study is distinct from previous research in this field in Taiwan. Here, we assessed the potential negative impact of the 2002 tobacco tax scheme on tobacco-related and non-tobacco industries and the macro-economy. In accordance with Lee *et al*. (2005), the increase in the price of cigarettes from the new tax scheme would reduce consumption by 18 percent overall. Accordingly, we used the 18 percent reduced tobacco consumption in our computable general equilibrium (CGE) model to assess the potential impact of the reduction in cigarette consumption after the new tax scheme was implemented.

### Overview of the modeling approach

The multisector computable general equilibrium model (hereafter, the CGE model), as used here, disaggregated the market into 18 producing sectors, 18 consuming sectors, 1 household sector, 1 enterprise sector and the government. It could analyze the broader economic effects of the reduced cigarette consumption after the new tax scheme went into effect. The model followed the trade analysis research of Chou and King (1994). In this model, we assumed that the factor market and product markets were perfectly competitive, meaning that all the primary factors of production were perfectly mobile [26]. We solved the CGE model with nonlinear programming using the General Algebraic Modeling System package, version 2.50.

The standard steps in constructing and using CGE models are: (1) to build a benchmark equilibrium data set – the Social Accounting Matrix (SAM). The CGE is based on a Social Accounting Matrix of the economy. In practice, the benchmark data base is constructed from national accounts, input-output tables, family income and expenditures, trade and balance of payments, value-added data (capital income by industry and labor income by industry) and other government data sources; (2) to set the basic behavior of institutions and to choose a functional form (for model equations and the definition of the variables, refer to Devarajan, Lewis, and Robinson [26], 1991 and Chou and King [27], 1994); (3) to specify extraneous elasticity values and to determine parameter values through calibration. Calibration follows a deterministic approach to specifying the CGE model parameter values. We assumed that an economic equilibrium is observed in the presence of existing policies. The first task in performing a general equilibrium analysis is not to solve for equilibrium, but rather to use the observed equilibrium to solve for model parameters consistent with that observation. (Shoven and Whalley, 1992) [28]; (4) to conduct replication checking (including solving for model benchmark values and verifying *Walras' Law*); and (5) to simulate the policy simulation [29].

Details concerning the various sectors for the CGE model in this study are presented in Table 1. There are eighteen productive sectors: agriculture, tobacco leaves, minerals, tobacco (including cigarettes, processed tobacco, other tobacco products and by-products), electronics, transport equipment and transport services, machinery, as well as several manufacturers, including food manufacturing, chemical and intermediate materials manufacturing, warehousing, electricity (including gas and water), construction, retail trade, advertising services and other services. These sectors provide goods and services to firms and households and consist of two factors of production – labor and capital. Each sector produces a composite commodity that is distributed in domestic or foreign markets within the context of a constant elasticity of a transformation (CET) function which captures the imperfect transformation between the two types of products. It is assumed that producers maximize their revenue from sales, subject to their production technology. This is presented in the form of a two-level nested Leontief production function. Intermediate inputs are used based on a fixed proportion, i.e., the Leontief production function. All primary factors are combined using a Cobb-Douglas function (the production functions have the feature of exhibiting constant returns to scale or homogenous of degree one).

With the CGE model, it is generally assumed that consumers maximize the utility function subject to a budget constraint equaling the factor income less taxes to which remittances from abroad and governmental transfers are added. This income is allocated to savings, private consumption and direct taxes. Utility maximization is achieved using a constant elasticity of substitution (CES) function. Households are assumed to purchase optimal quantities of composite private goods, treating domestic and imported goods as imperfect substitutes.

The final demand is derived from the behavior of consumers, the government, enterprises and foreigners. Consumer expenditures are a function of prices and income simplified with the version of the Linear Expenditure System (LES). The government levies taxes on both production and consumption. Revenues are used to purchased goods and services, and income is distributed back to households and enterprises (so called current transfers). Government demand for final goods is defined in terms of fixed shares of aggregate real government spending on goods and services. The demand for new inventories is specified by fixed coefficients and time production. Aggregate nominal fixed investment equals total nominal investment minus the value of inventory accumulation. Similarly, sectoral capital accumulations are given by fixed shares, which are summed to one over all sectors. Investment is determined by savings and deleted from the last version.

### Sources of the data

The aggregated and secondary data in this study to analyze the economic effects of change in cigarette consumption were obtained from the government. The data collected from the input-output tables in Taiwan (see Table 1) were previously reported by the Directorate General of Budget, Accounting and Statistics (DGBAS), Executive Yuan, Republic of China, as published in the *1999 Input-Output Tables*, Taiwan Area, Republic of China [31]. The Input-Output table in 1999 shows the inter-industry linkage effects. For this study, the *National Accounts Data and Family Income and Expenditure *data were taken from the DGBAS (2000) [32,33]. The structure of national income and household income are both explained there.

In addition, data for the primary factors of production, i.e., labor force and capital stock, were used in this paper, and these were taken from the *Taiwan Statistical Data Book *[34], published by the Council for Economic Planning and Development, Republic of China and from *Trends in Multifactor for Productivity*, Taiwan Area, Republic of China [35], published by DGBAS in 2000.

Year 2000 data were used in the model. In principle, all published reports should have consistent general equilibrium conditions. However, there were inconsistencies. For example, the input-output tables and capital composition matrix for 1999 are the most recent data up to 2000; these connect the various production sectors. Hence, a number of adjustments were required to ensure that equilibrium conditions hold. We relied heavily on the Row-and-Column Sum adjustment method for these modifications [28].

## Results

### Effects of cigarette tax on the industrial and tobacco sectors

Using the CGE model with all 18 sectors, we find multiple effects from the cigarette tax increase. First, in the industrial sectors, the total value added coefficients and domestic output figures merely show a slight increase of 0.0002 percent and 0.0077 percent (nearly NT$1.612 billion), respectively (see Table 2). In stark contrast, in the tobacco products sector, as might be expected, the corresponding values show a significant decrease of 0.005 percent and 17.382 percent (nearly NT$ 4.923 billion), respectively. The most critical impact, therefore, is in the tobacco products sector. In terms of upstream materials, domestic output in the tobacco leaves sector drops by 0.783 percent (about NT$ 90 million), while that in the manufacturing sectors, or more specifically in food, chemical, and intermediate materials, increases by between 0.085 and 0.258 percent, thus reflecting the expected diversion and redistribution of resources from the relative factor prices changed in tobacco products and leaves market. What this all implies is that the higher tax on tobacco products should have a significant adverse effect on the value added coefficients and domestic output in the tobacco products sector but that it should definitively be beneficial to other manufacturing sectors.

Total factor income in the industrial sectors should increase by 0.0174 percent or about NT$1.569 billion, but with a factor income decrease of 17.387 percent (about NT$546 million), the tobacco sector emerges as the first to be impacted by the reduced cigarette consumption. The next greatest reduction in factor income is in the tobacco leaf sector (0.785 percent or NT$53 million). It is noteworthy, however, that at the outset, in all likelihood, other sectors should not be noticeably influenced by the tax increase. Although the tax increase should have a notable negative impact on tobacco products and leaves, it should be positive for the increasing initial total factor income on the other industry.

A change in the labor market can also be anticipated as cigarette consumption declines in response to the tax increase. In this regard, layoffs are likely to increase by a high 17.380 percent (about 4,230 workers) in the tobacco products sector but by only a much lower 0.779 percent (about 23 workers) in the tobacco leaves sector in the first year. Afterward, employment directly related to tobacco products and leaves should continue to decline as a result of reduced tobacco consumption. Conversely, and equally important, employment in the agriculture, food manufacturing, basic manufacturing, and chemical manufacturing sectors should increase, largely because money that would have been spent on tobacco products is probably spent on other goods and services. Thus, the tax increase can be expected to play a major role in a redistribution of labor resources.

### Effects of cigarette tax on macroeconomic structure and consumer welfare

Based on the simulation in this study, the 2002 cigarette tax scheme should result in an immediate reduction of NT$1.275 billion in GDP and NT$2.217 billion in total investment. But there is a considerable NT$1.017 billion counterbalance in household consumption (see Table 3) on account of an offset by increased consumption in other sectors coupled with an increase in household income; this cannot be overlooked. It becomes clearly apparent, therefore, that reduced tobacco consumption does not negatively impact domestic macroeconomics. The Equivalent Variation (EV) was applied in this research as a standard for weighing consumer benefits [28]. Here, the new tax scheme raised household consumption for an increase of NT$13.098 billion in EV. It follows that the new tax scheme provides numerous benefits to consumer welfare.

### Health benefits from the additional cigarette tax

A cigarette tax increase has the potential to substantially reduce the number of smokers, the amount of tobacco consumed and the costs curtailed from smoking-related illnesses, including premature death. In 2002, Levy and Wen predicted that under the new Taiwan tax scheme, by 2003, there would be a 2.5 to 5 percent drop in the smoking rate [36]. With 4.5 million smokers in Taiwan, this meant 112,500 to 225,000 would quit smoking as a result of the tax increase. Using the estimated epidemiological analysis reported by the World Bank [5], approximately 28,125 to 56,250 lives could be saved with the implementation of a new tax scheme in Taiwan. Taiwan's GDP per person in 2002 was NT$434,684 (US$1 = 34.6 Taiwan dollars) [32]. With 112,500 to 225,000 smokers quitting, the estimated health benefits are estimated at NT$1.222 billion (28,125 lives×NT$434,684) to NT$2.445 billion (56,250 lives×NT$434,684) in 2002.

## Discussion

### Model adequacy

The impact of a tobacco taxation policy is usually based on the adjustment and transmission process via industry and market. In this study, the CGE model was implemented to evaluate the inter-industry relationships within the tobacco market, factors market and intermediate input and income inflow distribution from economic institutes after the tobacco taxation policy. It seems convincing that the tax scheme would influence the structure of the tobacco industry, resource allocation and the redistribution of household income. More importantly, we utilized the CGE model to effectively calculate the causal relation and feedback information system.

Overall, applying the CGE model to quantify the impact of the new tobacco taxation policy on the tobacco industry has four advantages. First, both the macro and micro economic variables in our model estimate the influence of inter-sectors on macro and micro economies, while simultaneously appraising substantial changes in the whole tobacco industry and then predicting further possible developments. Second, our model precisely computes the spillover effects induced by the implementation of the tobacco taxation scheme through the inter-industry relationships from the empirical model. Third, our model includes the interaction between cigarette prices and quantity variables that influence industry resource reallocation after the tobacco taxation increase. It is not necessary to make the design and simulation of the tobacco taxation policy mechanisms difficult. Fourth, by combining the public sector's budget with CGE estimations, government authorities can better comprehend changes brought about by tobacco taxation policy and the causal feedback from cash flows resulting from household consumption, savings and income.

However, our CGE model has some limitations as well. First, the model fails to acknowledge the finite resource base in a real economy. It is not clear the extent to which tobacco industry resources transfer to other industry sectors and contribute to unemployment, how much land there is that is changed for other uses, how much smaller the labor force is, and how much less capital there is that is underinvested after a particular tobacco taxation policy. This could result from the effects of introducing to new cigarette taxation and its influence on inter-industry relationships.

Another weakness of our model lies in its failure to identify the absence of an explicit budgetary constraint for private households. This is because there is no linkage between the sources and uses of income. Thus, if factor returns fall, it is not reflected in reduced consumer expenditures. This limitation is most severe when the shock from the introduction of a tobacco taxation policy is substantial, resulting in income transfers between households with very different consumption patterns. The larger and more complex our model is, the greater the probability will be that inconsistencies exist.

### Data quality

In that the data we used to perform our estimations were gathered from official statistical publications, we feel justified to conclude that they adequately manifest their quality. Although the industry relationship tables and capital accumulation matrixes in 1999 are far fewer than those for the base year 2000, they are in agreement with the real economical conditions after repeated collaboration in our model. Our results also attest to the ideal quality of the official statistical data. The replication check and recalibration procedures were conducted repeatedly in order to maintain the validity of the CGE model applied in this paper. Although the results from the CGE model cannot be tested by some specific statistical indicators, we performed some simulation work that showed the validity of the estimated results from our CGE model.

Nevertheless, a problem could possibly exist with the 160 classified sectors in the industry relationship tables for 1999. Some of the sectors had been combined and calculated by government officials. It was impossible to rearrange them to their original form. This could have resulted in some biased empirical results. For example, the degree of impact that the introduction of the tobacco taxation scheme had on the tobacco sector is exhibited as insignificant because its value had been summed into the other special purpose crops. Consequently, the effect of the new taxation scheme on the tobacco sector could have been cancelled out or possibly mixed with the effects on other sectors. Based on the interactions among the different sectors, the more complex the sector classifications are, the closer the empirical results are to the real economy.

### Summary

In terms of low price elasticity of cigarette demand and low inter-industry linkages, a rise in cigarette prices caused by the tobacco control policy should only have a negligible negative effect on the domestic macroeconomic structure. Moreover, positive economic benefits have been greatly gained from the enhancement on overall consumer welfare due to the decreased cigarette consumption after the implement of a "Tobacco Health and Welfare Tax". Equally important, the increased tax should reduce the extremely high incidence of death from smoking-related illnesses and increase the overall health benefits to society. Consequently, the strong incentives on the part of the government to implement the cigarette tax scheme as a policy tool for tobacco control seem fully justified.

## Conclusion

The main purpose of this study was to analyze the new tobacco tax scheme, introduced in 2002, in terms of the effects it has on the economy and the health benefits it provides in Taiwan by using a CGE model. Based on the results from this model, it is evident that the 2002 tax increase immediately contributes to a NT$8.5 billion increase in government tax revenue but a NT$599 million reduction in factor income from the two tobacco sectors. These sectors could be economically motivated to diversify into other product sectors by being offered short-term, cross-subsidy incentives from the additional tax revenue. No significant impact should result in the transport service, warehousing, retail trade, or advertising services sectors.

In our simulation, the cigarette price increase had only a negligible impact on the overall economic structure. A reduction in cigarette consumption should lead to a slight decrease of around 0.013 percent in GDP. Based on the results, it can be expected that the tax increase should reduce cigarette consumption by 1.836 billion packs in the first year; it should also result in 28,125 to 56,250 lives being saved from tobacco-related illnesses, and total health insurance savings should amount to NT$1.222 to NT$2.445 billion. In all, these health benefits exceed the decrease in GDP. These benefits aside, the rise in cigarette prices should also lead to an NT$13.098 billion increase in household welfare. This could be interpreted as an impressive tenfold counter-effect against the decrease in GDP (about NT$1.275 billion).

## Competing interests

The author(s) declare that they have no competing interests.

## Authors' contributions

CYY and JML have contributed to designating the study, acquiring the data, conducting the statistical analysis, interpreting the empirical analysis and preparing the manuscript. SHC has contributed to preparing the manuscript and interpreting the empirical analysis. All authors have read and approved the final manuscript.

## Pre-publication history

The pre-publication history for this paper can be accessed here:



## Figures and Tables

**Table 1:** Mapping of 160 Sectors Input-Output (I/O) Matrix in 18 Sectors

Sector	Code	Descriptions
*Agriculture*	001~003, 005~012	paddy rice, common crops, fruits, hogs, vegetables, fisheries, forestry, livestock
*Tobacco leaves*	004	tobacco leaves, other special crops
*Minerals*	013~017	coal, crude oil, metallic minerals, salt
*Food manufacturing*	018~032	rice, flour, canned food, beverages, frozen foods, dairy products, slaughtering and by-products, sugar, misc. food products,
*Tobacco products*	033	tobacco (cigarettes, processed tobacco, other tobacco products, by- products)
*Basic manufacturing*	034~053	wood, fabrics (cotton, worsted, artificial, and knitted), leather, printing, paper, paper products, garments
*Chemical manufacturing*	054~065,068~070	chemical fertilizers, fibers, plastic medicines, basic industrial chemicals, misc. chemical manufacturers
*Intermediate material manufacture*	066~067,071~085	steel, non-metallic and metallic products, cement
*Electronics*	091,092,096~105	household electrical appliances, computer products, video and radio electronics, communication apparatuses, semi-conductors, optoelectronic products
*Transport equipment*	106~110	shipbuilding, motor vehicle, motorcycle
*Machinery*	086~090,093~095, 111~113	machinery, precision instruments
*Transport services*	125~130	railway, water, and air transportation
*Warehousing*	131	warehousing
*Electricity, Gas, Water*	114~116	electricity, gas, water
*Construction*	117~120	residential building construction, public works
*Retail trade*	121~123	wholesale, retail, international trade
*Advertising services*	143154	advertising services, radio, television
*Other services*	124,132~142,144~153,155~160	food and beverage, postal, telegram and telephone, finance, public administration, and other services

*Data source*: Directorate General of Budget, Accounting and Statistics (DGBAS), Executive Yuan, Republic of China. *The Report on 1999 Input-Output Tables*, Taiwan Area, Republic of China [30].

**Table 2:** Impact of the increase in cigarette tax on tobacco and related sectors through CGE model simulation. Figures are given as a percentage change.

Sector\Indicates	Value-added coefficient	Domestic output	Factor income	Labor
*Agriculture*	0	0.127	0.127	0.133
*Tobacco leaves*	-0.002	-0.783	-0.785	-0.779
*Minerals*	-0.001	0.06	0.059	0.065
*Food manufacturers*	0	0.258	0.258	0.264
*Tobacco products*	-0.005	-17.382	-17.387	-17.38
*Basic manufacturers*	-0.002	0.243	0.241	0.284
*Chemical manufacturers*	0	0.192	0.192	0.198
*Intermediate materials manufacturers*	0	0.085	0.085	0.091
*Electronics*	0.002	-0.123	-0.121	-0.115
*Transport equipment*	-0.001	-0.024	-0.025	-0.02
*Machinery*	-0.001	-0.021	-0.022	-0.016
*Transport services*	-0.001	0.013	0.012	0.018
*Warehousing*	0.002	0.02	0.022	0.028
*Electricity, Gas, Water*	0.004	0.061	0.065	0.071
*Construction*	-0.003	-0.063	-0.066	-0.06
*Retail trade*	-0.001	0.014	0.013	0.019
*Advertising services*	-0.001	27	0.026	0.032
*Other services*	0.001	0.013	0.014	0.02

***Total effect***	**0.0002**	**0.0077**	**0.0174**	-*

* In the static CGE model, the aggregate labor supply fix is assumed.

**Table 3:** Impact of the increase in cigarette tax on macroeconomic and consumer welfare. Total change is given in NT $ billion.

Variables\Change	Total	Percentage
Real GDP	-1.275	-0.013
Total investment	-2.217	-0.094
Household consumption	1.017	0.017
Equivalent Variation (EV)	13.098	-
